# Dignity in the care of older adults living in nursing homes and long-term care facilities

**DOI:** 10.12688/f1000research.126144.1

**Published:** 2022-10-24

**Authors:** Patrick Wachholz, Karla Giacomin

**Affiliations:** 1 Programa de Pós-Graduação em Pesquisa Clínca, Faculdade de Medicina de Botucatu, Universidade Estadual Paulista (Unesp), Botucatu, São Paulo, 18618687, Brazil; 2 Núcleo de Estudos em Saúde Pública e Envelhecimento, Fundação Oswaldo Cruz, Belo Horizonte, Minas Gerais, Brazil

**Keywords:** long-term care, older adults, dignity, aging

## Abstract

Depending on the fields and actors involved, dignity may involve, signify, and encompass different meanings. This fundamental right can be subjectively experienced and rooted in a person’s perception of being treated and cared for. Care refers to a set of specific activities combined in a complex life-sustaining network, including long-term Care, which involves various services designed to meet a person’s health or personal care needs. However, older residents’ human rights have been disrespected and widened the gaps between theory and practice regarding the precarious protection of their rights and dignity inside long-term facilities and nursing homes. This paper aims to discuss threats to dignity and elucidate some strategies to promote and conserve dignity in care, including the person-centered practice in long-term care. Some barriers to the dignity of older residents involve the organizational culture, restraints of time, heavy workload, burnout, and lack of partnership between the residents, their families, and the long-term care homes’ staff. Person-centered integrated care quality frameworks are core components of a good quality of care in these spaces in high-income countries. Unfortunately, the COVID-19 pandemic highlighted how weak long-term care policies were and demonstrated that much progress in the dignity of care in long-term care facilities and nursing homes is needed. In low- and middle-income countries, long-term care policies do not accompany the accelerated and intense aging process, and there are other threats, like their invisibility to the public sector and the prejudices about this service model. It’s urgent to create strategies for designing and implementing sustainable and equitable long- term care systems based on a person-centered service with dignity to everyone who needs it.

## Introduction

*“All human beings are born free and equal in dignity and rights. They are endowed with reason and conscience and must act towards one another in a spirit of brotherhood.*

*(Universal Declaration of Human Rights, 1948).*

Flagship value, dignity may involve, signify, and encompass different meanings depending on the fields and actors involved and expected reciprocity between them. For example, the perception of dignity for professionals and healthcare providers can sound different when compared to the perceptions of healthcare users, their families and policy makers. ^1^^,^^2^

Despite some controversial criticism on defining it, ^3^ dignity is a core concept that must be guaranteed to any human being, whatever their condition is. Some authors argue that dignity may be a link that explains the interchange between the promotion and protection of human rights and an individual’s health status. ^4^^,^^5^ Recently, a substantial body of literature reviewing and analyzing the concepts of ‘dignity’, ‘care with dignity’, and ‘dignified care’ have been published, ^6^^,^^7^ reinforcing that dignity is considered to be a fundamental right, subjectively experienced, and rooted in a person’s perception of being treated and regarded as essential and valuable to others. ^8^

Care refers to the provision of a set of specific activities combined to provide help, protection, or supervision in a complex life-sustaining network. It may involve distinct actors and actions, including self-care, caring for others, the caregiver, and the care recipient. ^9^ Of note, the increased demand for care by the older population occurs concomitantly with changes in its provision: a declining number of carers challenges the informal sector due to trends toward reduced family size, a higher proportion of single households, more opportunities for women in the labor market, and increasing migration rates and geographic separation between parents and children. ^10^

Long term care (LTC) involves services designed to provide what is necessary to reach personal care needs, or to maintain their daily lives during a short or long period, particularly for those with limited capacity for self-care because of a chronic illness, injury, physical, cognitive, or mental disabilities. ^11^ LTC includes assistance with activities of daily living (ADLs) and instrumental activities of daily living (IADLs), including dispensing and correct administration of medications, engaging in household chores and hobbies and self-care tasks.

In most of the countries, LTC provision involves a mix of formal (state, local authority and nongovernment) and informal (primarily unpaid family) provision – though there are wide differences in the balance between these sectors in the context of national cultures and welfare traditions. ^12^ In low- and middle-income countries, LTC is mostly provided informally (typically by family and friends), even for the most vulnerable and functionally-impaired older adults, despite the growth of public and private LTC services in many of these nations.

LTC services help people improve or maintain an optimal quality of life and physical functioning, including but not limited to help from third parties or assistive devices in various settings: in the community (adult daily service center); at home, from a home health agency, hospice, or family and friends; in facilities (nursing home or specialized infirmary); or other residential settings. ^13^ According to the World Health Organization, “LTC homes are living spaces for adults with significant health challenges” in accessing 24-hour nursing and personal care. ^14^

Despite global and regional initiatives in favor of strengthening strategies to promote respect and dignity of older adults, such as the Decade of Healthy Aging, ^15^ LTC facility residents do not seem to have the same priorities and guarantees as their counterparts. The disrespect for older residents’ human rights during the recent period of the COVID-19 pandemic, for example, widened the gaps between theory and practice regarding the precarious protection of their rights and dignity. ^16^

This paper aims to discuss threats to dignity in LTC facilities (LTCF) and nursing homes (NH) and elucidate some strategies to promote and conserve dignity in care, including the person-centered practice in LTC.

### Threats to dignity in LTC homes

Even though care is provided proficiently or technically competent, residents and family members may perceive it as lacking in dignity. The concept of dignity for the older people living in LTC homes relates to feelings of comfort, autonomy, meaning, interpersonal connection, hope, physical and spiritual state, and belonging, and is influenced by their social interactions and positively or negatively affected by others. ^1^^,^^17^

The Nordenfelt’s theoretical dignity model, developed within the Dignity and Older Europeans Project, ^18^ provides a comprehensive definition of dignity that is very useful to understand how fostering a culture of dignity may have an impact on older residents. It distinguishes between intrinsic and contingent value in four concepts as follows: “ *Dignity of merit*: related to a person’s formal or informal status in society; *Dignity as moral stature*: linked to self-respect and dependent on the conduct of the individual; *Dignity of identity*: attached to the person’s identity as a human being, which can be altered by others or external events; *Dignity of Menschenwürde*: a German word meaning innate or inner dignity that is afforded all humans.” ^18^

The first three concepts of dignity described by Nordenfelt can vary and often depend on individuals’ conduct, autonomy, integrity, and the people they interact. In the context of aging or illness, dignity of identity is probably the most important of the previous concepts. ^1^ In contrast, Menschenwürde’s dignity deals with innate dignity, which we all possess equally. ^19^

Despite some differences in the causes of admission to NH and LTCF in low- and high-income countries, it is common for a large proportion of residents to have a significant reduction in their cognitive and functional abilities, depending on third parties to perform ADLs and IADLs. According to some authors, dependency affects their dignity (of identity), because it can reduce their control and choice. ^17^^,^^19^

Rigid or inflexible technical and organizational routines depersonalize care in LTC homes, depriving residents of expressing their opinions and desires. Due to time constraints, resources, and caregivers’ propensity for task-oriented care, the depersonalization of care often compromises the resident’s dignity, who is forced to “obey” mealtimes, hygiene standards, and continence, participation in social activities, and sometimes even control over one’s belongings. ^1^^,^^17^^,^^19^ Even when residents have their cognition and desire for autonomy preserved, a tension may emerge when organizations decide to maintain a ‘risk-free environment’ by forcing staff and residents to obey rules that limits autonomy and control. ^1^^,^^19^

Caregivers’ communication with older residents (or other workers) about themselves or their peers can also threaten the dignity of LTC, even when a resident has impaired communication skills. Using potentially stigmatizing or ageist labels, diminutives or nicknames when referring to a resident is highly undesirable, as well as publicly exposing personal information due to hearing impairment in collective settings. ^1^^,^^17^^,^^18^

Dignity in LTC must always be linked to values of personhood and unique identity and disaggregated from the use of any form of physical or chemical restraints. The “zero tolerance” culture of abuse against the older resident must be an organizational dogma understood and practiced by all staff, including volunteers.

The right to privacy includes concepts of respect for the dignity of identity also in the promotion of assistance during the control and rise of continence, respecting the resident’s desire for service provided by caregivers of the same sex, for example. The right to privacy includes reducing exposure to the body or assistive devices (such as prostheses or urinary catheters).

Even in environments where economic deprivation can substantially impact access to inputs and food, ensuring frequent, healthy, and palatable meals must be essential. Disregarding food consumption preferences, especially during the approximation periods after entering an LTC home, can significantly impact the perception of dignity and outcomes related to weight loss, sarcopenia, and, consequently, worsening of functional abilities.

### Dignity-conserving care in LTC

Some authors previously suggested best practices for compliance related to resident dignity, ^20^ focusing on requirements that include respecting care needs, maximizing the dining experience, living in a secure facility, participating in activities, and respecting residents’ personal space. ^21^ Best practices may include, for instance, assuring residents preferences related to personal appearance are consistently honored, developing a policy for selective use of clothing protectors during meals and an environment to ensure that direct staff can comfortably assist with feeding, besides addressing residents by their names and providing meaningful activities considering the residents’ abilities and past interests.

It is important to highlight that dignity of older residents cannot be promoted without reciprocal partnership between them, their family and the LTC homes’ staff. ^1^ Despite previous studies found that organizational culture, restraints of time, heavy workload and burnout have been cited as barriers to a dignified care, ^6^^,^^22^^,^^23^ providers must make sure that the care and treatment they provide ensure people’s dignity, including having privacy when they need and want it, treating them as equals and providing the support they might need, including involving them in the local community activities. ^24^

Person-centered integrated care (PC-IC) quality frameworks are core components of a good quality of care in LTC. It is possible to build a 4-stage goal-oriented PC-IC process, ^17^ including (a) personalizing goal settings, (b) care planning aligned with goals, (c) care delivery according to plan, and (d) evaluation of goal attainment. ^25^ A theoretical framework for person-centered practice in long-term care (PeoPLe) is another example of providing a comprehensive guide to empirical inquiry, education, and practice development in LTC homes, serving as a low-threshold starting point for practice development. ^26^

In addition to constructs in the framework of person-centered practice, previous authors have found significant associations between self-rated health, mobility, and dementia and perceptions of dignity and well-being. ^27^^–^^29^

Using a modified Delphi process to prioritize essential ‘dignity-conserving care markers’, Thompson and colleagues ^17^ found the following practices to be good markers: staff make residents feel valued as a person; staff are compassionate in providing care; residents can trust staff; staff do not make residents feel like a burden to others; residents are able to make choices in their everyday life; ^17^ assistance with hygiene and personal matters is adequate and sensitive; there is freedom to complain without fear of repercussions; staff does not talk about residents in front of other residents; the personal space of the residents and the need for privacy are respected; efforts are made to make residents feel safe.

The COVID-19 pandemic highlighted long-known weaknesses and shortcomings, which were continually postponed in terms of public and social relevance in its resolutions. It demonstrated that we need to make much progress in the dignity of care in LTC. The challenge of caring for older residents is particularly felt in low- and middle-income countries, where the development of LTC policies does not accompany the accelerated and intense aging process in a context of marked social and gender inequality. ^13^

However, given the lack of a national LTC policy, this gap’s most explicit practical expression is the reduced and heterogeneous offer of institutional care in these countries.

When considering the provision of comprehensive and person-centered care in LTCF, fundamental aspects must be considered by the workers, family, and managers. Among these aspects, the life project (i.e., the direction that the individual wants to take according to their beliefs) stands out; preferences and their scale of values; the story of life, with which we can get to know the person more deeply and pay close attention. Finally, individualized service plans facilitate the detection of needs, turning LTCFs into units of conviviality closer to a domestic environment in terms of organization, schedules, and spaces.

According to the person-centered practice framework, significant associations were found between attitudes of staff, thriving in the indoor-outdoor-mealtime environment, and perceptions of dignity and well-being. This approach targets the attitudes of staff and the care environment, which could be used when designing interventions to promote dignity and well-being. ^27^

In conclusion, dignity in the LTC goes through the recognition of its need and the support of public policies that, in addition to monitoring, promote more significant knowledge about the reality of the care offered. This also means the confrontation of prejudice about this service model ^27^ and the urgent creation of strategies for designing and implementing sustainable and equitable LTC systems ^28^ that ensure a person-centered service with dignity to everyone who needs it.

## Data Availability

No data are associated with this article.
