# Dignity in the care of older adults living in nursing homes and long-term care facilities

**DOI:** 10.12688/f1000research.126144.3

**Published:** 2023-10-30

**Authors:** Patrick Wachholz, Karla Giacomin

**Affiliations:** 1 Programa de Pós-Graduação em Pesquisa Clínca, Faculdade de Medicina de Botucatu, Universidade Estadual Paulista (Unesp), Botucatu, São Paulo, 18618687, Brazil; 2 Núcleo de Estudos em Saúde Pública e Envelhecimento, Fundação Oswaldo Cruz, Belo Horizonte, Minas Gerais, Brazil

**Keywords:** long-term care, older adults, dignity, aging

## Abstract

Depending on the fields and actors involved, dignity may involve, signify, and encompass different meanings. This fundamental right can be subjectively experienced and rooted in a person’s perception of being treated and cared for. Care refers to a set of specific activities combined in a complex life-sustaining network, including long-term Care, which involves various services designed to meet a person’s health or personal care needs. However, older residents’ human rights have been disrespected and widened the gaps between theory and practice regarding the precarious protection of their rights and dignity inside long-term facilities and nursing homes. This paper aims to discuss threats to dignity and elucidate some strategies to promote and conserve dignity in care, including the person-centered practice in long-term care. Some barriers to the dignity of older residents involve the organizational culture, restraints of time, heavy workload, burnout, and lack of partnership between the residents, their families, and the long-term care homes’ staff. Person-centered integrated care quality frameworks are core components of a good quality of care in these spaces in high-income countries. Unfortunately, the COVID-19 pandemic highlighted how weak long-term care policies were and demonstrated that much progress in the dignity of care in long-term care facilities and nursing homes is needed. In low- and middle-income countries, long-term care policies do not accompany the accelerated and intense aging process, and there are other threats, like their invisibility to the public sector and the prejudices about this service model. It’s urgent to create strategies for designing and implementing sustainable and equitable long- term care systems based on a person-centered service with dignity to everyone who needs it.

## Introduction

*“All human beings are born free and equal in dignity and rights. They are endowed with reason and conscience and must act towards one another in a spirit of brotherhood.*

*(Universal Declaration of Human Rights, 1948).*

Despite some controversial criticism, ^1^ dignity remains a concept that is difficult to define, measure, and apply to healthcare, mainly because it intersects with other terms such as pride, self-respect, quality of life, well-being, hope, self-esteem, autonomy, respect, empowerment, and communication. ^2^ Yet, it is a core concept that must be guaranteed to any human being, whatever their condition is.

Dignity, a flagship value, may involve, signify, and encompass different meanings depending on the fields and actors involved and expected reciprocity between them. For example, the perception of dignity for professionals and healthcare providers can sound different when compared to the perceptions of healthcare users, their families and policymakers. ^3^^,^^4^

Ostaszkiewicz *et al.* ^2^ state that international reports identify a lack of attention to the dignity of older people in care homes and hospitals. Other authors argue that dignity may be a link that explains the interchange between promoting and protecting human rights and an individual’s health status. ^5^^,^^6^ Recently, a substantial body of literature has been published reviewing and analyzing the concepts of ‘dignity’, ‘care with dignity’, and ‘dignified care’, ^7^^,^^8^ reinforcing that dignity is considered to be a fundamental right, subjectively experienced, and rooted in a person’s perception of being treated and regarded as essential and valuable to others. ^9^

Care refers to providing specific activities combined to provide help, protection, or supervision in a complex life-sustaining network. It may involve distinct actors and actions, including self-care, caring for others, the caregiver, and the care recipient. ^10^

The increased demand for care occurs concomitantly with demographic shifts toward population aging (notably faster in low- and middle-income countries) and with changes in its provision: a declining number of carers (due to reduced family size), a higher proportion of single households, more opportunities for women in the labor market, and increasing migration rates and geographic separation between parents and children challenge the lack of support and financial subsidies for caring for their family members. ^11^

Long-term care (LTC) involves services designed to provide what is necessary to reach personal care needs, or to maintain their daily lives during a short or long period, particularly for those with limited capacity for self-care because of a chronic illness, injury, physical, cognitive, or mental disabilities. ^12^ LTC includes assistance with activities of daily living (ADLs) and instrumental activities of daily living (IADLs), including dispensing and correct administration of medications, engaging in household chores and hobbies and self-care tasks.

In most countries, LTC provision involves a mix of state, for-profit and voluntary (third sector – formal or informal provision), though there are wide differences in the balance between these sectors in the context of national cultures and welfare traditions. ^13^ In low- and middle-income countries, LTC is mostly provided by the third sector (typically by family and friends), even for the most vulnerable and functionally impaired older adults, despite the growth of public and private LTC services in many of these nations.

LTC services help people improve or maintain an optimal quality of life and physical functioning, including but not limited to help from third parties or assistive devices in various settings: in the community (adult daily service center); at home, from a home health agency, hospice, or family and friends; in facilities (nursing home or specialized infirmary); or other residential settings. ^14^ According to the World Health Organization, “LTC homes are living spaces for adults with significant health challenges” in accessing 24-hour nursing and personal care. ^15^

Despite global and regional initiatives in favor of strengthening strategies to promote respect and dignity of older adults, such as the Decade of Healthy Aging, ^16^ LTC facility residents do not seem to have the same priorities and guarantees as their counterparts. The disrespect for older residents’ human rights during the recent period of the COVID-19 pandemic, for example, widened the gaps between theory and practice regarding the precarious protection of their rights and dignity. ^17^

This paper aims to discuss threats to dignity in LTC facilities (LTCF) and nursing homes (NH) and elucidate some strategies to promote and conserve dignity in care, including the person-centred practice in LTC. In addition to reviewing factors that threaten dignity in LTC homes (like inflexible routines, reduced perceptions of the importance of dignity, ageism, and low concern for communication skills/right to privacy), we will present attitudes and care that preserve it in this context (like person-centred care), examples of observational studies and interventionist investigations, and real-life studies with interventions and practical recommendations to support the care that favors and fosters dignity.

### Threats to dignity in LTC homes

Even though care is provided proficiently or technically competent, residents and family members may perceive it as lacking dignity. The concept of dignity for older people living in LTC homes relates to feelings of comfort, autonomy, meaning, interpersonal connection, hope, physical and spiritual state, and belonging, and is influenced by their social interactions and positively or negatively affected by others. ^1^^,^^18^

The Nordenfelt’s theoretical dignity model, developed within the Dignity and Older Europeans Project, ^19^ provides a comprehensive definition of dignity that is very useful to understand how fostering a culture of dignity impact on older residents. It distinguishes between intrinsic and contingent value in four concepts as follows: “ *Dignity of merit*: related to a person’s formal or informal status in society; *Dignity as moral stature*: linked to self-respect and dependent on the conduct of the individual; *Dignity of identity*: attached to the person’s identity as a human being, which others or external events can alter; *Dignity of Menschenwürde*: a German word meaning innate or inner dignity that is afforded all humans.” ^19^

The first three concepts of dignity described by Nordenfelt can vary and often depend on individuals’ conduct, autonomy, integrity, and the people they interact. In the context of aging or illness, dignity of identity is probably the most important of the previous concepts. ^1^ In contrast, Menschenwürde’s dignity deals with innate dignity, which we all possess equally. ^20^

Despite some differences in the causes of admission to NH and LTCF in low- and high-income countries, it is common for many residents to significantly reduce their cognitive and functional abilities, depending on third parties to perform ADLs and IADLs. According to some authors, dependency affects their dignity (of identity), because it can reduce their control and choice. ^18^^,^^20^

Rigid or inflexible technical and organizational routines depersonalize care in LTC homes, depriving residents of expressing their opinions and desires. Due to time constraints, resources, and caregivers’ propensity for task-oriented care, the depersonalization of care often compromises the resident’s dignity, who is forced to “obey” mealtimes, hygiene standards, and continence, participation in social activities, and sometimes even control over one’s belongings. ^1^^,^^18^^,^^20^

According to Kitwood, ^21^ the ‘medical model’ produces bad care practices and a range of interactions between care staff and persons living with dementia (including those living in LTCF) that detract from a person’s personhood. What Kitwood ^21^ defined as ‘malignant social psychology’ is often unknowingly embedded in the care habits of formal care settings. The author concludes that identifying the care staff’s observed behaviors detracts from an individual’s personhood and highlights those behaviors that enhance an individual’s personhood. But even when residents have their cognition and desire for autonomy preserved, tension may emerge when organizations decide to maintain a ‘risk-free environment’ by forcing staff and residents to obey rules that limits autonomy and control. ^1^^,^^20^

Particularly among residents living with dementia, multiple studies suggested that stigmatization, labeling, and objectivation were found to be related to dignity violation. These authors found that undignifying aspects of care are characterized by unsuccessful processes of acknowledging and conciliating with the changing person with dementia. ^22^

Caregivers’ communication with older residents (or other workers) about themselves or their peers can also threaten the dignity of LTC, even when a resident has impaired communication skills. Using potentially stigmatizing or ageist labels, diminutives or nicknames when referring to a resident is highly undesirable, as well as publicly exposing personal information due to hearing impairment in collective settings. ^1^^,^^18^^,^^19^

Dignity in LTC must always be linked to values of personhood and unique identity and disaggregated from using of any form of physical or chemical restraints. The “zero tolerance” culture of abuse against older residents must be an organizational dogma understood and practiced by all staff, including volunteers.

The right to privacy includes concepts of respect for the dignity of identity also in the promotion of assistance during the control and rise of continence, respecting the resident’s desire for service provided by caregivers of the same sex, for example. The right to privacy includes reducing exposure to the body or assistive devices (such as prostheses or urinary catheters).

Even in environments where economic deprivation can substantially impact access to inputs and food, ensuring frequent, healthy, and palatable meals must be essential. Disregarding food consumption preferences, especially during the approximation periods after entering an LTC home, can significantly impact the perception of dignity and outcomes related to weight loss, sarcopenia, and, consequently, worsening of functional abilities.

### Dignity-conserving care in LTC

Few intervention studies have examined care that maintains dignity. A qualitative study including in-depth interviews with residents of LTC homes in Japan found dignity to be related to nursing care facilities, and the nursing care system involves teams/organizations from elements of the staff side at the individual level. ^23^

Some authors suggested best practices for compliance related to resident dignity, ^22^ focusing on requirements that include respecting care needs, maximizing the dining experience, living in a secure facility, participating in activities, and respecting residents’ personal space. ^24^ Best practices may include, for instance, assuring residents’ preferences related to personal appearance are consistently honored, developing a policy for selective use of clothing protectors during meals, and an environment to ensure that direct staff can comfortably assist with feeding, besides addressing residents by their names and providing meaningful activities considering the residents’ abilities and past interests.

It is important to highlight that the dignity of older residents cannot be promoted without reciprocal partnership between them, their families and the LTC homes’ staff. ^3^ Despite previous studies found that organizational culture, restraints of time, heavy workload and burnout have been cited as barriers to a dignified care, ^7^^,^^25^^,^^26^ providers must make sure that the care and treatment they provide ensure people’s dignity, including having privacy when they need and want it, treating them as equals and providing the support they might need, including involving them in the local community activities. ^27^

A Canadian study with Dignity Therapy, a psychotherapeutic intervention for patients near to the end of life, found 75% of participants reporting an enhanced sense of dignity. ^28^

Person-centred integrated care (PC-IC) quality frameworks are core components of a good quality of care in LTC. It is possible to build a 4-stage goal-oriented PC-IC process, ^18^ including (a) personalizing goal settings, (b) care planning aligned with goals, (c) care delivery according to plan, and (d) evaluation of goal attainment. ^29^ A theoretical framework for person-centred practice in long-term care (PeoPLe) is another example of providing a comprehensive guide to empirical inquiry, education, and practice development in LTC homes, serving as a low-threshold starting point for practice development. ^30^

In addition to constructs in the framework of person-centred practice, previous authors have found significant associations between self-rated health, mobility, and dementia and perceptions of dignity and well-being. ^31^^–^^34^

Using a modified Delphi process to prioritize essential ‘dignity-conserving care markers’, Thompson and colleagues ^18^ found the following practices to be good markers: staff make residents feel valued as a person; staff are compassionate in providing care; residents can trust staff; staff do not make residents feel like a burden to others; residents are able to make choices in their everyday life; ^18^ assistance with hygiene and personal matters is adequate and sensitive; there is freedom to complain without fear of repercussions; staff does not talk about residents in front of other residents; the personal space of the residents and the need for privacy are respected; efforts are made to make residents feel safe.

The COVID-19 pandemic highlighted long-known weaknesses and shortcomings, continually postponed in terms of public and social relevance in its resolutions. It demonstrated that we need to make much progress in the dignity of care in LTC. The challenge of caring for older residents is particularly felt in low- and middle-income countries, where the development of LTC policies does not accompany the accelerated and intense aging in a context of marked social and gender inequality. ^14^

However, given the lack of a national LTC policy, this gap’s most explicit practical expression is the reduced and heterogeneous offer of institutional care in these countries.

When considering the provision of comprehensive and person-centred care in LTCF, fundamental aspects must be considered by the workers, families, and managers. Among these aspects, the life project (i.e., the direction the individual wants to take according to their beliefs) stands out; preferences and their scale of values; the story of life, with which we can get to know the person more deeply and pay close attention. Finally, individualized service plans facilitate the detection of needs, turning LTCFs into units of conviviality closer to a domestic environment in terms of organization, schedules, and spaces.

According to the person-centred practice framework, significant associations were found between the attitudes of staff, thriving in the indoor-outdoor-mealtime environment, and perceptions of dignity and well-being. This approach targets the attitudes of staff and the care environment, which could be used when designing interventions to promote dignity and well-being. ^31^

Practical recommendations for LTC homes care providers must consider including PCC-IC, respecting care needs, personal space, and the right to privacy, including care of people with dementia, discussing options and interventions for dying with dignity, and decision-making with dignity.

In conclusion, dignity in the LTC goes through the recognition of its need and the support of public policies that, in addition to monitoring, promote more significant knowledge about the reality of the care offered. This also means the confrontation of prejudice about this service model ^31^ and the urgent creation of strategies for designing and implementing sustainable and equitable LTC systems ^32^ that ensure a person-centred service with dignity to everyone who needs it. Considering the urgency of fostering long-term care systems in aging societies by involving public opinion and policymakers, countries can design and implement LTC systems centered on human rights and integrated into the overall health system, encouraging multisectoral collaboration to strengthen national and regional efforts in dignity and healthy aging. ^35^

## Data Availability

No data are associated with this article.
