# Supplementary material for: Dignity in the care of older adults living in nursing homes and long-term care facilities
Source: F1000Res. 2023 Oct 30;11:1208. Originally published 2022 Oct 24. [Version 3] doi: 10.12688/f1000research.126144.3 (PMC10904933; doi:10.12688/f1000research.126144.3)
Supplement: Peer review history

# Reviewer report: Reviewer response for version 3

**DOI:** 10.5256/f1000research.157963.r247407

**Published:** 2024-02-21

**Authors:** Elaine Webster

**Affiliations:** 1 University of Strathclyde, Glasgow, Scotland, UK

Thank you for the opportunity to review this article, ‘Dignity in the care of older adults living in nursing homes and long-term care facilities’. It addresses a topic which remains highly significant. The paper’s stated aim, to ‘discuss threats to dignity in LTC facilities (LTCF) and nursing homes (NH) and elucidate some strategies to promote and conserve dignity in care’ is valuable. As an opinion piece, including a non-systematic review, it provides an accessible introduction and serves to keep this critical issue on the agenda.  

 The following comments, relating to clarity and evidence base, provide suggestions to further enhance the article: 

 The authors may wish to clarify that in ‘discussing’ threats to dignity, the paper both shares some of the authors’ own views – such as on restraint, responding to incontinence, and food provision, which are not linked to specific existing sources – and provides an overview of some of the threats to, and means of respecting, dignity that have been identified in research.  

 In terms of existing research, one of the key sources in the discussion of dignity is somewhat dated – the Nordenfelt and Edgar source which dates from 2005. Some further explanation of why this remains the most useful conceptualisation, almost 20 years later, may strengthen the piece.  

 It may add to the discussion if the authors were to unpack the ‘controversial criticism’ referred to in the opening line. The authors might also consider rewording this sentence as the controversy referred to seems incongruent with the statement that dignity ‘remains’ difficult to define.  

 The authors start from the basis that ‘dignity’ encompasses a range of meanings and might be perceived differently depending on one’s perspective/experience. This is indeed demonstrated in literature. It would thereby be helpful to see more precise use of the term ‘dignity’ within the article itself. In my view, the terminology of ‘dignity’ and ‘rights’ is used quite loosely whereas these can be distinct if overlapping terms/concepts/policy and practice frameworks. There is reference to ‘dignity’ as a fundamental right and to ‘dignity and rights’ as different categories. Another concept also mentioned is personhood. At the same time, it is not always clear whether the authors are fundamentally interested in ‘dignity’ specifically, or rather in the related idea and practice of person-centred long-term care. The authors may thereby wish to consider whether the focus could be sharpened in this respect. 

 Some additional evidence could be given for the point relating to low- and middle-income countries in the introduction and in the section on threats to dignity in LTC homes. In the introduction, for example, the closest sources, cited in notes 11 and 13, do not appear to speak to the issue of LTC in low- and middle-income countries.  

 It would also be helpful to have more precision regarding the sources for the following points by adding additional reference detail, as it is not currently clear whether these terms/findings are reported in the source cited (in note 2) or are original findings of that source: ‘dignity remains a concept that is difficult to define, measure, and apply’, and that dignity ‘intersects with other terms such as pride, self-respect, quality of life, well-being, hope, self-esteem, autonomy, respect, empowerment, and communication.’

Is the topic of the opinion article discussed accurately in the context of the current literature?

Yes

Are arguments sufficiently supported by evidence from the published literature?

Partly

Are all factual statements correct and adequately supported by citations?

Partly

Are the conclusions drawn balanced and justified on the basis of the presented arguments?

Yes

Reviewer Expertise:

Human rights law, Meaning and role of concept of ‘dignity’, Dignified care and human rights-based approach in nurse education.

I confirm that I have read this submission and believe that I have an appropriate level of expertise to confirm that it is of an acceptable scientific standard, however I have significant reservations, as outlined above.

# Reviewer report: Reviewer response for version 3

**DOI:** 10.5256/f1000research.157963.r238176

**Published:** 2024-02-15

**Authors:** Wendy Van der Geugten

**Affiliations:** 1 University of Humanistic Studies, Utrecht, The Netherlands

Abstract:

 This article discusses dignity threatening care activities within long-term care and care conventions and activities fostering dignity of residents, with a focus on person-centred care.

 Overall remark:

- I think the authors want to be sensitive for context, mostly in referring to differences between high and low-/middle-income countries. Although in general the awareness of these socio-political context is appreciated, I do not understand the importance for repeating these statements/comparisons in light of the overall argument of this article. This is also partly about referring to the COVID-19 pandemic, it seems that both context indicate the importance of improving and prioritizing LTC but I do not follow the line with the main topic of the article (dignity conserving and person-centred care).
  - When the authors indeed want to make a point of LTC within low-/middle income countries and how dignity and person-centred care needs to improve, then they have to point this out more clearly with substantiated with studies (or a lack of research into this topic).
- I was a little confused on the placements of the parts below, which deviates from the line of argumentation above concerning person-centred care and personhood, as well missing references. The authors might consider these aspects of (in)dignity in care to be placed earlier in the article before working towards their statement.
  - “The right to privacy includes concepts of respect for the dignity of identity also in the promotion of assistance during the control and rise of continence, respecting the resident’s desire for service provided by caregivers of the same sex, for example. The right to privacy includes reducing exposure to the body or assistive devices (such as prostheses or urinary catheters)”
  - “ Even in environments where economic deprivation can substantially impact access to inputs and food, ensuring frequent, healthy, and palatable meals must be essential. Disregarding food consumption preferences, especially during the approximation periods after entering an LTC home, can significantly impact the perception of dignity and outcomes related to weight loss, sarcopenia, and, consequently, worsening of functional abilities.”
- Is this correct? “Few intervention studies have examined care that maintains dignity”. – in the following text I miss studies into Dignity Therapy, and the ground work of Chochinov into dignity-conserving care practices, there is only one sentence referring to this body of work. It might be interesting to see if this research can help your article.
- I think that the argument that person-centred care is improving dignity of residents needs to be fully clear before presenting studies into PC-IC under ‘dignity-conserving care’. The authors might firstly present all studies into dignity and dignity-conserving care, than present the theory of Kitwood and PC-IC studies, to bring along the reader in line of the argument and makes it easier to compare both.

References needed (beside the above mentioned topics):

-  "In low- and middle-income countries, LTC is mostly ...”
- “….. LTC facility residents do not seem to have the same priorities and guarantees as their counterparts”.
- “When considering the provision of comprehensive and person-centred care in LTCF, fundamental aspects must be considered by the workers, families, and managers. Among these aspects, the life project (i.e., the direction the individual wants to take according to their beliefs) stands out; preferences and their scale of values; the story of life, with which we can get to know the person more deeply and pay close attention. Finally, individualized service plans facilitate the detection of needs, turning LTCFs into units of conviviality closer to a domestic environment in terms of organization, schedules, and spaces”.

Is the topic of the opinion article discussed accurately in the context of the current literature?

Partly

Are arguments sufficiently supported by evidence from the published literature?

Partly

Are all factual statements correct and adequately supported by citations?

Partly

Are the conclusions drawn balanced and justified on the basis of the presented arguments?

Partly

Reviewer Expertise:

Dignity, long-term care, COVID-19, palliative care, dementia, spiritual care

I confirm that I have read this submission and believe that I have an appropriate level of expertise to confirm that it is of an acceptable scientific standard, however I have significant reservations, as outlined above.

# Reviewer report: Reviewer response for version 2

**DOI:** 10.5256/f1000research.144106.r198053

**Published:** 2023-09-21

**Authors:** Sergej Kmetec

**Affiliations:** 1 Department of Health Sciences, University of Maribor, Maribor, Slovenia

The article "Dignity in the Care of Older Adults Living in Nursing Homes and Long-Term Care Facilities" provides a comprehensive overview of the challenges and strategies related to preserving the dignity of older adults in long-term care settings. Given the growing ageing population globally and the issues the COVID-19 pandemic has exacerbated, the topic is of utmost importance. The article is well-structured, informative, and addresses a critical issue. However, there are some areas where it could be improved:

1. **Clarity and Structure**: The article's structure is clear, with distinct sections discussing dignity in LTC, threats to dignity, and dignity-conserving care. However, it might benefit from a more explicit introduction section that provides an overview of the key points to be discussed. This can help readers grasp the main objectives and structure of the paper more easily.
2. **Citation and Evidence**: While the article does mention previous studies and models related to dignity in LTC, it would be strengthened by citing specific research studies or real-world examples to support the points being made. Incorporating concrete evidence can enhance the credibility of the arguments presented.
3. **Language and Style**: The language used in the article is generally clear and professional. However, some sentences could be made more concise and easier to understand. Additionally, some of the language might be considered technical, so providing clear explanations for specialized terms or concepts would make the article more accessible to a broader readership.
4. **Practical Recommendations**: While the article discusses the importance of person-centred care and dignity-conserving practices, it could benefit from offering more practical recommendations or case studies demonstrating how these strategies can be implemented effectively in real-world LTC settings. This would provide readers with actionable insights.
5. **Global Perspective**: The article briefly discusses the differences in LTC policies between high-income and low- to middle-income countries. Expanding on this topic and providing a more in-depth analysis of the challenges and potential solutions in various global contexts would add depth to the discussion.
6. **Conclusion**: The conclusion does a good job of summarizing the key points discussed in the article. However, it could be strengthened by emphasizing the urgency of addressing these issues and providing a clear call to action for policymakers, healthcare providers, and researchers.
7. **Terminology and grammatical suggestions** **: **Please use person-centred care instead of "person-centered care" throughout the manuscript because it is appropriate terminology from the field of person-centred care.

In conclusion, the article provides a valuable exploration of the importance of dignity in LTC and the challenges older adults face in such settings. The article could benefit from clearer structure, more concrete evidence, and practical recommendations for improving the dignity of care in LTC facilities to enhance its impact. Nevertheless, it significantly contributes to the ongoing discourse on this critical issue and encourages further research and action.

Is the topic of the opinion article discussed accurately in the context of the current literature?

Yes

Are arguments sufficiently supported by evidence from the published literature?

Partly

Are all factual statements correct and adequately supported by citations?

Yes

Are the conclusions drawn balanced and justified on the basis of the presented arguments?

Partly

Reviewer Expertise:

Nursing, Person-centred care, Palliative care, Care of older people, Dementia, Systematic review, Quantitative, Qualitative and Mixed method research

I confirm that I have read this submission and believe that I have an appropriate level of expertise to confirm that it is of an acceptable scientific standard, however I have significant reservations, as outlined above.

# Response

**Published:** 2023-10-22

**Authors:** Patrick Wachholz

**Affiliations:** Sao Paulo State University, Brazil

**Reply to the reviewer.**

 The authors agree with the reviewer's comments and consider that the proposed changes provide greater robustness and clarity to the description of this opinion article.

 In the introduction, the authors included, after the objectives, a description of the key points to be discussed, helping readers understand the manuscript's structure and record its main messages.

 Practical recommendations were added to the final comments, allowing readers to consider gaps in the literature when developing new investigations and applying models and practices that strengthen care with dignity in LTC homes. We include mentions of international, observational, qualitative, and intervention studies in end-of-life care, highlighting strategies effectively adopted in the real world.

 We revised the language and style of the entire manuscript, correcting dubious sentences and making them more precise and concise.

 Finally, we adopted the recommendation to use the term 'person-centered care,' as suggested.

# Reviewer report: Reviewer response for version 2

**DOI:** 10.5256/f1000research.144106.r163874

**Published:** 2023-02-27

**Authors:** Neil H. Chadborn

**Affiliations:** 1 School of Medicine, University of Nottingham, Nottingham, UK

The authors have made amendments and have addressed concerns raised. This is now revised article is now suitable for publication and I hope achieves an international readership.

Is the topic of the opinion article discussed accurately in the context of the current literature?

Yes

Are arguments sufficiently supported by evidence from the published literature?

Yes

Are all factual statements correct and adequately supported by citations?

Yes

Are the conclusions drawn balanced and justified on the basis of the presented arguments?

Yes

Reviewer Expertise:

Public health research and health and social care services research.

I confirm that I have read this submission and believe that I have an appropriate level of expertise to confirm that it is of an acceptable scientific standard.

# Reviewer report: Reviewer response for version 1

**DOI:** 10.5256/f1000research.138524.r154087

**Published:** 2022-11-15

**Authors:** Neil H. Chadborn

**Affiliations:** 1 School of Medicine, University of Nottingham, Nottingham, UK

This article discusses dignity, an important topic in health and social care, which can be overlooked in health research literature. This is partly because it is difficult to quantify but also because it may not be seen as a 'health outcome'. However, similar to person-centred care, dignity is a crucial element of care as expressed by residents and family. 

   

 The introduction, following the abstract, starts from an ambiguous point, with dignity potentially being different to different people and between different groups of people. This appears at odds with the quote about dignity and rights as being universal concepts. While, clearly there may be different attitudes or priorities with respect to dignity. The next paragraph develops the approach that there is a ‘core concept’ (at least) for the meaning of dignity, so I wonder whether this could be incorporated into the first paragraph so the article does not start with such an open definition.

 “Of note, the increased demand for care by the older population occurs concomitantly with changes in its provision” – clarity could be improved by stating what scale of change is being discussed – it appears that demographic shift is being discussed, however it could be read as the care of a cohort of people changes as they age. Further, this demographic change may relate to USA and is different other countries? Also, I question the term ‘informal sector’ – if this mainly means family members, I would argue that this group is not a ‘sector’ in an economic sense. My point is that if someone is not receiving a service, then they may be supported by family members; I don’t believe they would be described as ‘receiving long-term care provided by the informal sector’.

 Also, I have same concerns with this para “In most of the countries, LTC provision involves a mix of formal (state, local authority and nongovernment) and informal (primarily unpaid family) provision – though there are wide differences in the balance between these sectors in the context of national cultures and welfare traditions.” I would argue that family provided care should not be included in the category of ‘receiving care service’. I would define the different sectors, then, as: voluntary (third sector – informal or formal), for-profit, and state. The reason that I labour this point is that individuals (particularly in ‘Western’ cultures) do express different views when they are receiving care from family or friends compared to receiving ‘a service’; particularly for intimate care, individuals may feel that this compromises their dignity, whereas receiving care from a careworker or volunteer from an NGO is likely to be seen as protecting their dignity. This point may be Western-centric and may not apply in different cultural contexts, but I believe the distinction remains important. In fact maybe dignity cannot be described independently of familial duty.

 “Rigid or inflexible technical and organizational routines depersonalize care in LTC homes, depriving residents of expressing their opinions and desires. Due to time constraints, resources, and caregivers’ propensity for task-oriented care, the depersonalization of care often compromises the resident’s dignity, who is forced to “obey” mealtimes, hygiene standards, and continence, participation in social activities, and sometimes even control over one’s belongings” For this paragraph, I think it would be helpful addition to quote the work of Tom Kitwood on malignant social environments (Dementia Reconsidered, 1997).

 I think it would help clarity of the article to state that discussion is focused on long term care facilities – rather than care in own home (also included in the term long-term care). 

 While the authors emphasise protecting, promoting and conserving dignity, it may be worth touching on where there may be exceptions or trade-offs; particularly for safety of the individual, care staff or co-residents (including protection from outbreak of COVID-19). 

 Specific comments:

- Abstract: “long-term facilities” should be long-term care facilities
- Intro “Flagship value, dignity may involve, signify, and encompass different meanings” for readability amend to: Dignity, as a flagship value, may involve…

Check formatting of reference 4 – should be Mann, J. “Dignity and Health: The UDHR’s Revolutionary First Article.” Health and Human Rights, vol. 3, no. 2, 1998, pp. 30–38. JSTOR, https://doi.org/10.2307/4065297. Accessed 14 Nov. 2022.

Is the topic of the opinion article discussed accurately in the context of the current literature?

Yes

Are arguments sufficiently supported by evidence from the published literature?

Yes

Are all factual statements correct and adequately supported by citations?

Yes

Are the conclusions drawn balanced and justified on the basis of the presented arguments?

Yes

Reviewer Expertise:

Public health research and health and social care services research.

I confirm that I have read this submission and believe that I have an appropriate level of expertise to confirm that it is of an acceptable scientific standard, however I have significant reservations, as outlined above.

# Response

**Published:** 2023-02-06

**Authors:** Patrick Wachholz

**Affiliations:** Sao Paulo State University, Brazil

We very much appreciate the reviewer's comments and recommendations. We have included your suggestions in this new version, corrected minor errors, and revised the bibliographic references.

 We agreed with the reviewer that it was necessary to adapt the first paragraph to start the manuscript with a less open definition of dignity.

 We agree that the term "informal sector" may convey the wrong message to the audience and have amended this paragraph accordingly.

 We have included a mention of Tom Kitwood's work on malign social psychology and its impact on the attitudes and practices of caregivers in the formal sector.

 Likewise, we corrected the title so that the manuscript focuses on long-term care facilities. 

 We agree that during the COVID-19 pandemic, adaptations and exceptions to practice were necessary to reduce the odds of outbreaks and ensure the safety of care homes, their workers, and residents.
